# Early Staphylococcal Biofilm Formation on Solid Orthopaedic Implant Materials: *In Vitro* Study

**DOI:** 10.1371/journal.pone.0107588

**Published:** 2014-10-09

**Authors:** Hironobu Koseki, Akihiko Yonekura, Takayuki Shida, Itaru Yoda, Hidehiko Horiuchi, Yoshitomo Morinaga, Katsunori Yanagihara, Hideyuki Sakoda, Makoto Osaki, Masato Tomita

**Affiliations:** 1 Department of Orthopedic Surgery, Graduate School of Biomedical Sciences, Nagasaki University, Nagasaki, Japan; 2 Department of Laboratory Medicine, Graduate School of Biomedical Sciences, Nagasaki University, Nagasaki, Japan; 3 Division of Medical Devices, National Institute of Health Sciences, Tokyo, Japan; LSU Health Sciences Center School of Dentistry, United States of America

## Abstract

Biofilms forming on the surface of biomaterials can cause intractable implant-related infections. Bacterial adherence and early biofilm formation are influenced by the type of biomaterial used and the physical characteristics of implant surface. In this *in vitro* research, we evaluated the ability of *Staphylococcus epidermidis*, the main pathogen in implant-related infections, to form biofilms on the surface of the solid orthopaedic biomaterials, oxidized zirconium-niobium alloy, cobalt-chromium-molybdenum alloy (Co-Cr-Mo), titanium alloy (Ti-6Al-4V), commercially pure titanium (cp-Ti) and stainless steel. A bacterial suspension of *Staphylococcus epidermidis* strain RP62A (ATCC35984) was added to the surface of specimens and incubated. The stained biofilms were imaged with a digital optical microscope and the biofilm coverage rate (BCR) was calculated. The total amount of biofilm was determined with the crystal violet assay and the number of viable cells in the biofilm was counted using the plate count method. The BCR of all the biomaterials rose in proportion to culture duration. After culturing for 2–4 hours, the BCR was similar for all materials. However, after culturing for 6 hours, the BCR for Co-Cr-Mo alloy was significantly lower than for Ti-6Al-4V, cp-Ti and stainless steel (*P*<0.05). The absorbance value determined in the crystal violet assay and the number of viable cells on Co-Cr-Mo were not significantly lower than for the other materials (*P*>0.05). These results suggest that surface properties, such as hydrophobicity or the low surface free energy of Co-Cr-Mo, may have some influence in inhibiting or delaying the two-dimensional expansion of biofilm on surfaces with a similar degree of smoothness.

## Introduction

Solid biomaterials with particular characteristics, such as high biocompatibility or corrosion resistance, are now being implanted in the human body more frequently for a wide range of purposes. However, implant-related infection is generally the most common serious complication and the risk of surgical site infection (SSI) increases when a foreign material is present [1]. When bacteria adhere to and proliferate on the biomaterial surface, the bacteria produce extracellular polymeric substances, primarily polysaccharides, which mediate cell-to-cell adhesion and form a biofilm. The biofilm enveloping the bacteria can protect them from the immune system. Moreover, the presence of biofilm changes gene expression, alters growth rate, and decreases susceptibility to antibiotics [2]–[6], so implant-related infection is extremely difficult to treat [7]–[9]. Various methods have been devised to prevent implant-related infections, including techniques to sterilize the surgical site and instruments, and the use of highly sterile operating rooms. However, these infections still occur today in 0.2–17.3% of orthopaedic surgery [10]–[12]. Therefore, research investigating the formation of biofilms on biomaterials is critically important from the clinical perspective.

The process of biofilm formation is generally thought to be a two-step model. Firstly, bacteria rapidly adhere to the biomaterial surface by means of physicochemical interactions (van der Waals forces, gravitational forces, electrostatic repulsion, and ionic and dipole interactions). Secondly, the bacteria proliferate and accumulate to form multilayered cell clusters on the surface through molecular and cellular interactions [13], [14]. Most implant-related infections are caused by *Staphylococcus* species [15]–[17]. The skin commensal organism *Staphylococcus epidermidis* (*S. epidermidis*) has been recognized as the preeminent and important medical pathogen in orthopaedic implant-related infections. It is particularly capable of adhering to and aggregating on biomaterial surfaces and can form biofilms on many biomaterials [18], [19]. Arciola et al demonstrated that multiple instances of resistance to antibiotics were more frequent among polysaccharides producing the *S. epidermidis* strain [3]. Research studies have shown that polysaccharide intercellular adhesin (PIA) plays an important role in biofilm formation and development along with genetic factors such as *ica ADBC* [19]–[23]. However, the detailed mechanism of this process has yet to be determined because of the complex combination of numerous other factors related to the bacteria, the *in vivo* environment and the use of artificial materials.

The solid biomaterials used for clinical purposes must be biocompatible and have a high resistance to wear, fracture and corrosion. Depending on their application, biomaterials can be made of just a few kinds of materials standardized by the International Organization for Standardization (ISO) and the American Society for Testing and Materials (ASTM). Oxidized Zirconium-Niobium alloy (Oxinium) was commercialized as a new biomaterial in Japan in 2008. This alloy forged from zirconium and niobium is permeated with oxygen at a high temperature, with only 5 µm of the surface changed to zirconium ceramic. As a result, Oxinium exhibits a low level of abrasion on sliding surfaces characteristic of a ceramic and has the strength of a metal. Oxinium also contains almost no toxic metals [24].

Recently, numerous factors related to the artificial solid biomaterials themselves, such as chemical structure, surface roughness, hydrophilicity, Z potential and surface free energy, have been identified as influencing bacterial adherence and early biofilm formation [25]–[33]. Although the evidence about the relationship between biomaterial and early phase of biofilm formation in previous studies is inconsistent, some previous reports have highlighted a relatively strong relationship between biofilm formation and surface roughness [30]–[32]. The rougher surface provides a wider area for bacterial adherence, multiplication and biofilm formation [33], [34]. However, there have been no studies into the effects of surface characteristics on bacterial biofilm formation apart from roughness. Therefore, in order to accurately compare the biofilm formation ability of the various biomaterials, we must eliminate this bias.

Several investigative methods have been established to evaluate the development of biofilms on the surface of biomaterials. Methods to directly examine biofilm formation include fluorescence microscopy (FM) [35], scanning electron microscopy (SEM) [36]. With these forms of image analysis, we can directly observe and enumerate the number of bacteria. Confocal laser scanning microscopy (CLSM) is a newly developed, valuable method for morphological observation of biofilm [37]–[39]. Indirect methods applicable for estimating biofilm density include viable cell count (VCC) after sonication [40], ATP-bioluminescence (ATP) [41], trypsin treatment [42] and crystal violet (CV) assay [43]. The VCC method is the most basic and conventional method for counting viable bacterial and the CV assay assesses the total amount of biofilm, including dead cells and extracellular polymeric substances. The percentage of surface covered by a biofilm is calculated as the biofilm coverage rate (BCR) [44]. The method for measuring BCR can estimate the growth of the biofilm using the time course, as well as assessing its two-dimensional expansion on non-translucent biomaterials without disrupting it.

In this *in vitro* study, we used BCR, CV assay and VCC to quantify the amount of biofilm formed by *S. epidermidis* and to compare its ability to form such biofilms on the surfaces of five types of solid biomaterials with a similar degree of smoothness. We have discovered no previous research focusing on the biofilm formation ability of different biomaterials, including Oxinium, which eliminates the influence of surface roughness.

## Materials and Method

### Specimen preparation

We prepared circular specimens (12 mm in diameter, 6 mm thick) from Oxinium (ASTM F2384), cobalt-chromium-molybdenum alloy (Co-Cr-Mo) (ASTM F75 high carbon), titanium alloy (Ti-6Al-4V) (ASTM F136), pure titanium (cp-Ti) (ASTM F67) and stainless steel (SUS316L) (ASTM F138) that are actually used in clinical practice. Original materials were obtained from Smith & Nephew Orthopaedics Inc. (Memphis, TM, USA) and Kakushin Surgical Instruments Co.Ltd. (Shizuoka, Japan). The five kinds of test specimen were progressively polished using a basic lapping machine (Doctorlap ML-180SL, Maruto Co.Ltd., Tokyo, Japan) with polishing compounds, a polishing cloth and a diamond slurry (Maruto Instrument Co. Ltd., Tokyo, Japan; 1 µm particle diameter).

### Surface characterization

Micrographs of the specimen disk surfaces were obtained using a field emission scanning electron microscope (SEM: JSM 6610LV, JEOL, Tokyo, Japan). The surface morphology and roughness of the specimens were measured by means of a 3D measuring laser microscope (OLS4000, Shimadzu, Tokyo, Japan) with a cut-off value (λc) of 80 µm at room temperature. Three readings were made of each surface on three random samples, and the average roughness (Ra) and mean roughness profile depth (Rz) were used to determine the roughness of the specimens. The initial contact angles of the surface of each specimen to deionized water (Milli-Q, EMD Millipore, Billerica, MA, USA) and diiodomethane (Wako Pure Chemical Industries Ltd. Osaka, Japan) were measured by the drop method using an automated contact angle measurement device (DSA30, Krüss GmbH, Hamburg, Germany) on each of three randomly selected specimens at room temperature (25°C). Prior to contact angle determination, all specimens were equilibrated with ethanol. The total surface free energy (γ^t^) and its polar (γ^p^) and disperse (γ^d^) components were calculated from the contact angles of deionized water and diiodomethane according to the Owen's (1) and Young's equation (2) [45]. 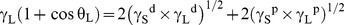
(1)

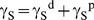
(2)


Where θ is the measured contact angle, γ_L_ is the surface free energy of the reference liquid, γ_L_ = γ_L_^d^+γ_L_^p^. γ_L_^d^ and γ_L_^p^ are the dispersive and polar components of surface free energy of the reference liquids, respectively. γ_S_^d^ and γ_S_^p^ are the dispersive and polar components of surface free energy of the solid surface, respectively. The contact angle θ is a measurable parameter. When two liquids with known γ_L_^d^ and γ_L_^p^ are used, γ_S_^d^ and γ_S_^p^ can be obtained by solving the two simultaneous equations. The total surface free energy of the solid (γ_S_) is the sum of γ_S_^d^ and γ_S_^p^. Deionized water (γ_L_^d^ = 21.8 mJ/m^2^, and γ_L_^p^ = 51.0 mJ/m^2^) and diiodomethane (γ_L_^d^ = 37.0 mJ/m^2^, and γ_L_^p^ = 26.4 mJ/m^2^) were used as the reference liquids [46]–[48].

### Experimental design

PIA-producing *S. epidermidis* strain RP62A (American Type Culture Collection [ATCC] 35984, American Type Culture Collection, Manassas, VA, USA) was grown overnight in Trypticase Soy Broth (TSB: Becton Dickinson Biosciences, Franklin Lakes, NJ, USA) at 37°C. The culture was diluted into TSB the following day at a ratio of 1: 10 and incubated for 3 hours to create a bacterial suspension of 1×10^5^ CFU/mL (logarithmic growth: Optical Density [OD] _600_ = 0.2; pH 7.0). Olson et al. investigated the superior adherence ability of PIA-producing *S. epidermidis* on biomaterial surfaces [23]. The test specimens were subjected to ultrasonic cleaning and autoclaving and then 200 µL of the bacterial suspension was dropped onto the specimens at room temperature and incubated for 60 minutes. The specimens were rinsed twice with phosphate-buffered saline (PBS: Sigma-Aldrich St Louis, MO, USA; pH 7.0) to remove non-adherent and loosely adherent cells, and transferred into fresh TSB medium for culturing (culture duration: 2 hours, 4 hours and 6 hours).

The morphology of the biofilms on the different specimens after 6 hours culture was assessed by SEM. The biofilm was fixed with glutaraldehyde (2.5% v/v) in a 0.1 M cacodylate buffer (0.1 M Na-cacodylate trihydrate in H_2_O, pH 7.4) for 4 hours at 4°C. The specimens were washed twice in the cacodylate buffer for 20 minutes followed by rinsing with H_2_O for 1 minute and biofilm was then post-fixed in 1% OsO_4_ for 2 hours at 4°C. The specimens were dehydrated with graded ethanol (50%, 70%, 80%, 90%, 95% and 99.5% v/v) for 10 minutes at each interval and dried using a freeze-dryer (ID-2, Engineering Co. Ltd., Mito, Japan). Finally, the biofilms were sputter-coated with platinum palladium using an ion-sputter (JFC-1600, JEOL, Tokyo, Japan), and viewed with a SEM at an accelerating voltage of 15 kV.

BCR measurements were performed as described previously [44]. The specimen surfaces were fixed with ethanol for 1 minute after which they were air dried and then stained with 0.5% crystal violet (Sigma-Aldrich, MD, USA) for 5 minutes. In order to remove the excess unbound dye, the specimens were then washed with distilled water and dried. The growth formation of the biofilm in the horizontal direction was observed using a digital optical microscope (VHX-100; Keyence, Osaka, Japan) and the percentage of the surface covered by bacteria was calculated as the biofilm coverage rate (BCR) [44], [49]. Images with ×450 full color photographs of a random nine locations on each specimen were obtained and converted to gray-scale images with Paint Shop Pro 8 (Corel Co., Ltd., Eden Prairie, MN, USA). The BCRs were measured using the Scion Image software package (Scion Co., Ltd., Frederick, MD) [50] and the BCR value of nine areas were averaged for each specimen.

The total amount of biofilm was assessed using a CV assay. The biofilm formed after 6 hours of culturing was fixed with ethanol and dried for 5 minutes. The fixed biofilms were stained with 0.5% crystal violet (Sigma-Aldrich, MD, USA) for 5 minutes. The excess unbound dye was removed by washing the specimens with distilled water. After thorough air drying, the specimens were transferred into sterile conical tubes (Falcon, BD Biosciences, Franklin Lakes, NJ, USA) filled with 5 mL of PBS. The tubes were vortexed at full speed for 3 minutes and then placed in an ultrasonic bath and sonicated for 5 minutes at 120 W to release the biofilm attached to the biomaterial. After an additional vortex step, the specimens were removed. The remaining suspensions were plated in triplicate in 96-well microtiter plate and the absorbance values were measured at an optical density of 570 nm using a microplate reader (Infinite F200 PRO, Tecan, Männedorf, Switzerland).

The VCCs in the suspension were counted using the standard plate count method. The specimens with biofilm incubated for the same 6 hours were each placed in sterile conical tubes containing 5 ml PBS. In order to remove the biofilm from the specimen, these tubes were vortexed for 3 minutes, sonicated for 5 minutes, and vortexed again for 3minutes. The solution containing the biofilm was transferred into another sterile conical tube and diluted with PBS. The number of viable bacteria in the biofilm was determined by counting the colony-forming units (CFUs) with a Compact Dry TC culture kit (Nissui Pharmaceutical Co., Ltd., Tokyo, Japan), after which the bacterial density (CFU/ml) was calculated. As well as using uniform conditions for the bacteria, the five kinds of specimens were treated at the same time, and the experiments themselves were repeated using a uniform procedure to eliminate the effect of environmental factors.

### Statistical analysis

The mean and standard deviation of the topographic parameters of the specimens (n = 6), contact angles (n = 12), BCR (n = 7), OD values (n = 10), and the VCC (n = 12) were analyzed for the different materials using SPSS 10.0 statistical software (SPSS Inc., Chicago, IL, USA). Statistical analysis was performed using one-way analysis of variance (one-way ANOVA), multiple comparison tests and the Tukey-Kramere and Bonferroni/Dunn multiple comparison test for *post hoc* analysis. The value of statistical significance was set at *P*<0.05.

## Results

**Figure 1:**
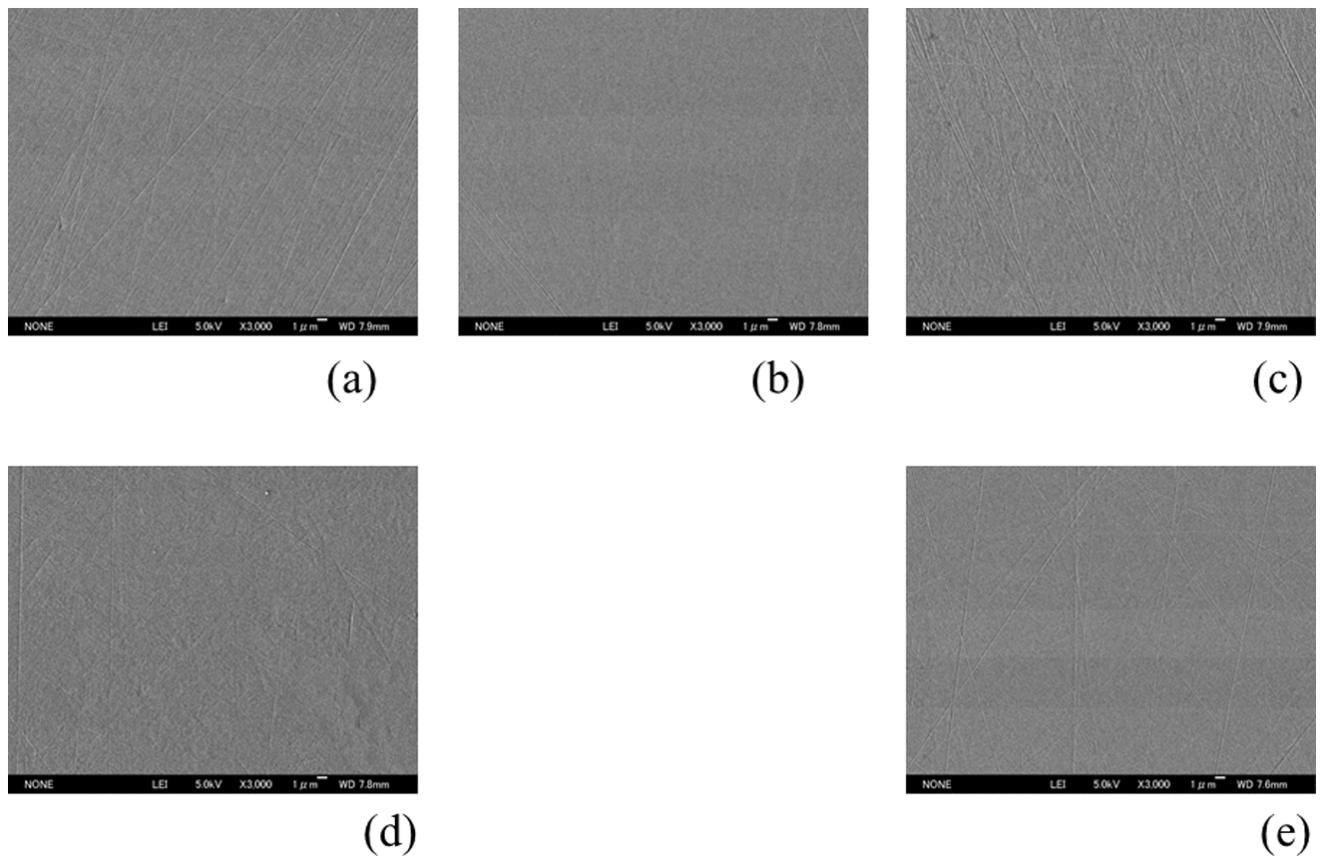
SEM micrographs. Although a few polishing micro-traces and marks were observed, all specimens had a generally featureless and smooth surface. Oxinium (a), Co-Cr-Mo (b), Ti-6Al-4V (c), cp-Ti (d), stainless steel (e) Original magnification ×3000 (Scale bar  = 1 µm)

Figure 1 shows SEM images of the prepared specimen surface. Although there are some fine polishing micro-traces and marks homogeneously distributed over the samples, all specimens were observed to be generally featureless with a smooth surface topography. The mean surface roughness parameters for each type of specimen are shown in Table 1. All specimens had comparatively smooth surfaces and recorded low average roughness (Ra<10 nm). The contact angles and surface free energies were shown in Table 2. The surface of Co-Cr-Mo had the highest water contact angle, followed by cp-Ti, stainless steel and Ti-6Al-4V. Oxinium yielded the lowest water contact angle. A greater water contact angle means a more hydrophobic surface. The total surface free energy of Co-Cr-Mo, which is composed of a low dispersive component, is relatively lower than that of the other biomaterials.

**Table 1:** Surface roughness.

	Roughness (nm)	
	Ra	Rz
Oxinium	6.3(1.9)^b^^,^ e	47.6 (10.1)^b^^,^ e
Co-Cr-Mo	1.9 (0.8)^a^^,^ c ^,^ d	14.2 (7.4)^a^^,^ c ^,^ d
Ti-6Al-4V	6.7 (1.1)^b^^,^ e	50.8 (14.6)^b^^,^ d ^,^ e
cp-Ti	5.3 (1.2)^b^^,^ e	34.9 (7.2)^b^^,^ c ^,^ e
stainless steel	1.2 (0.4)^a^^,^ c ^,^ d	9.2 (0.8)^a^^,^ c ^,^ d

Data were expressed as a mean (standard deviation (SD)).

Ra: arithmetic mean of the departures of the roughness profile from the profile center-line.

Rz: average distance between the highest peak and the lowest valley.

a : *P*<0.01 compared to Oxinium.

b : *P*<0.01 compared to Co-Cr-Mo.

c : *P*<0.01 compared to Ti-6Al-4.

d : *P*<0.01 compared to cp-Ti.

e : *P*<0.01 compared to stainless steel.

**Table 2:** Contact angles and Surface free energies.

	Contact angle (degree)				
	Water	Diiodomethane	γ_S_ d (mJ/m^2^)	γ_S_^p^ (mJ/m^2^)	γ_S_ (mJ/m^2^)
Oxinium	69.0 (3.9)^b^^,^ d ^,^ e	37.8 (1.1)^b^^,^ d ^,^ e	34.3	9.6	43.9
Co-Cr-Mo	107.3 (5.2)^a^^,^ c ^,^ d ^,^ e	49.7 (1.2)^a^^,^ c ^,^ d ^,^ e	28.4	9.7	38.1
Ti-6Al-4V	71.7 (0.3)^b^^,^ d ^,^ e	36.5 (0.8)^b^^,^ d ^,^ e	35.6	7.8	43.4
Cp-Ti	96.9 (6.6)^a^^,^ b ^,^ c	41.1 (1.0)^a^^,^ b ^,^ c	39.9	0.1	40.0
stainless steel	90.4 (2.3)^a^^,^ b ^,^ c	38.7 (1.7)^a^^,^ b ^,^ c	39.5	0.9	40.4

Contact angle data were expressed as a mean (standard deviation (SD)). Surface free energies (γ_S_) were calculated from the mean value of the contact angles of water and diiodomethane.

a : *P*<0.01 compared to Oxinium.

b : *P*<0.01 compared to Co-Cr-Mo.

c : *P*<0.01 compared to Ti-6Al-4.

d : *P*<0.01 compared to cp-Ti.

e : *P*<0.01 compared to stainless steel.

**Figure 2:**
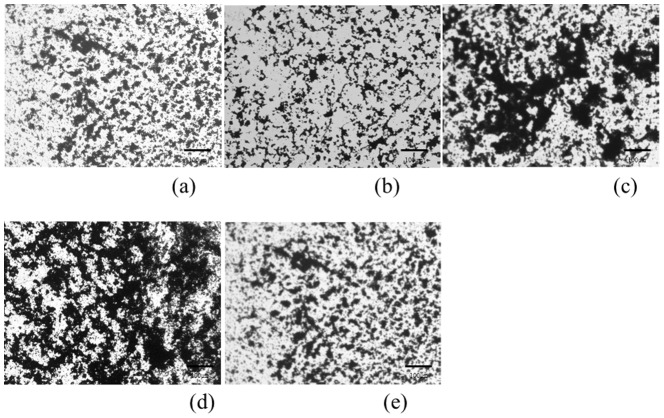
Digital optical micrographs. Biofilm incubated for 6 hours were stained with 0.5% crystal violet (black area). Oxinium (a), Co-Cr-Mo (b), Ti-6Al-4V (c), cp-Ti (d), stainless steel (e) Original magnification ×450 (Scale bar  = 100 µm)

**Figure 3:**
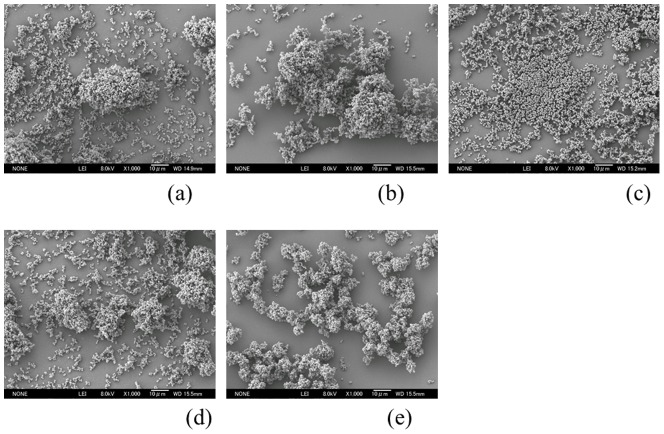
SEM images of biofilm. Oxinium (a), Co-Cr-Mo (b), Ti-6Al-4V (c), cp-Ti (d), stainless steel (e) Original magnification ×1000 (Scale bar  = 10 µm)

**Figure 4:**
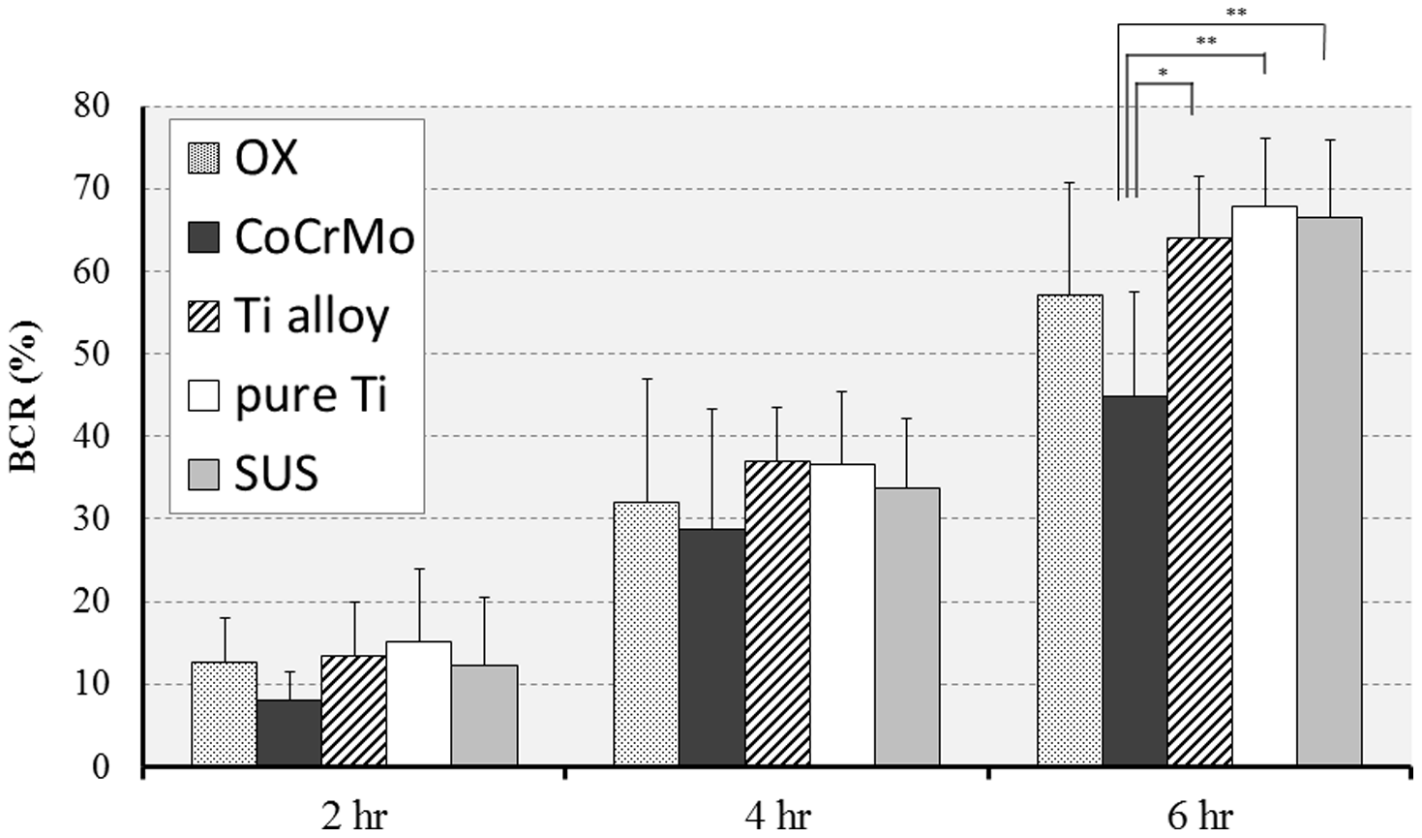
Biofilm coverage rate (BCR). Mean and standard deviation are shown. *p<0.05, **p<0.01

**Figure 5:**
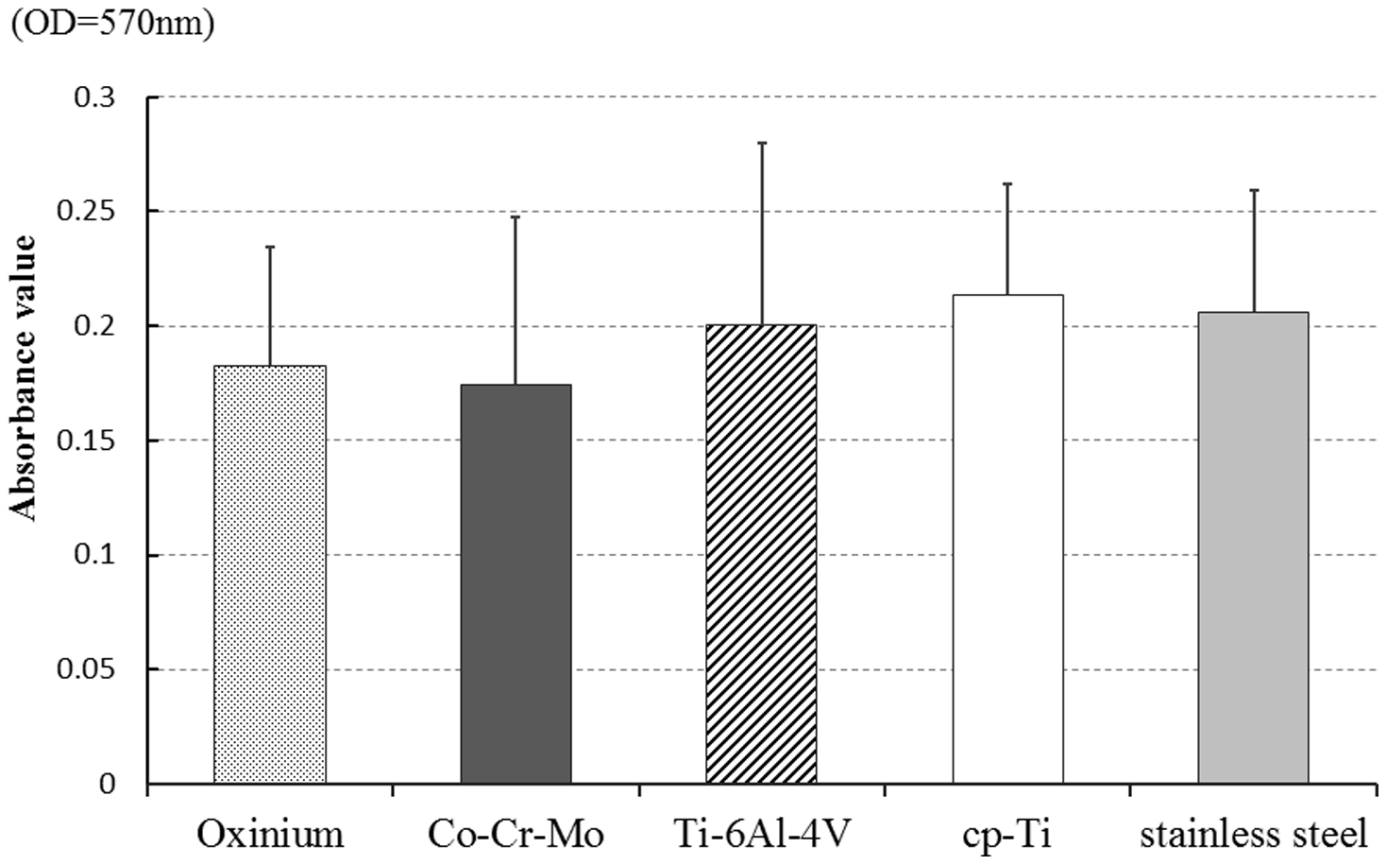
Absorbance value of crystal violet (CV) assay after 6 hours incubation. Mean and standard deviation are shown.

**Figure 6:**
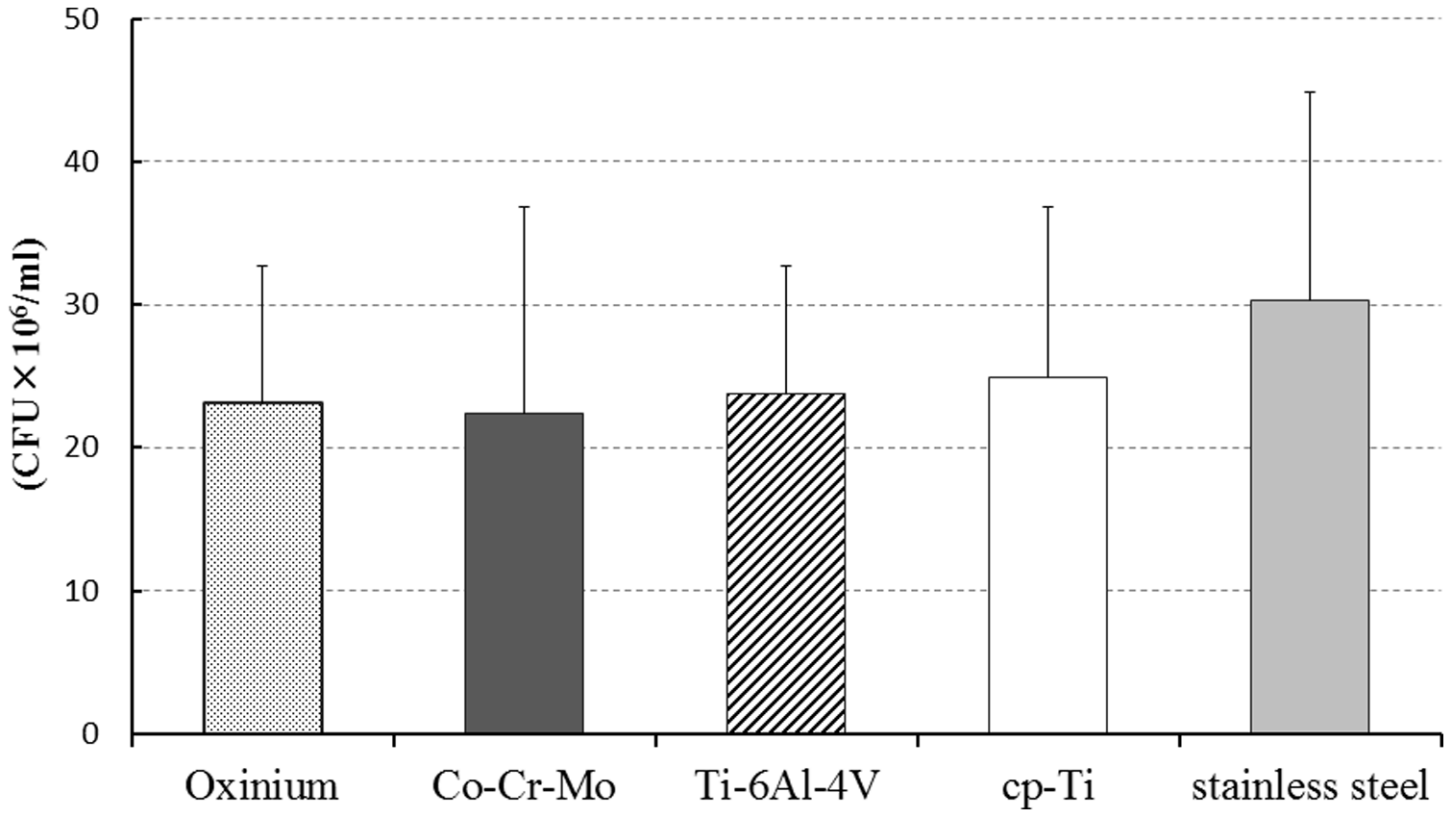
Colony forming units as determined by viable cell count (VCC) after 6 hours incubation. Mean and standard deviation are shown.

Biofilms formed on all of the specimens. Digital microscopic images of the biofilm after 6 hours culture is shown in Figure 2(a)∼(e). These images demonstrate that a wide area was covered by the stained biofilm on the surface of Ti-6Al-4V and cp-Ti. Observation using SEM revealed that the bacteria on Co-Cr-Mo was aggregated and more tightly colonized than for Oxinium, Ti-6Al-4V and cp-Ti (Figure 3(a)∼(e)). Biofilm colonies on the surface of Oxinium, Ti-6Al-4V and cp-Ti tended to be scattered and horizontally spread. The BCR rose as the culture duration increased (Figure 4). After culturing for 2 hours, the BCR was an average of 12.6±5.4% for Oxinium, 8.0±3.6% for Co-Cr-Mo, 13.4±3.3% for Ti-6Al-4V, 15.2±6.1% for cp-Ti and 12.2±4.4% for stainless steel. Therefore, there was no significant difference in BCR between the materials. After culturing for 4 hours, the BCR was higher than after 2 hours for all test specimens. Similarly to the findings after 2 hours culturing, no statistically significant differences were observed. After culturing for 6 hours, Co-Cr-Mo had the lowest BCR and there were statistically significant differences between the BCR of Co-Cr-Mo (44.8±12.6%) and that of Ti-6Al-4V (64.1±7.3%), cp-Ti (67.8±8.3%) and stainless steel (66.6±9.4%) (*P*<0.05). During 2–4 hours of culturing, the biofilms on all the biomaterials grew at a similar pace to the increase in BCR. Conversely, between 4–6 hours of culturing, the BCR developed gradually on Co-Cr-Mo compared to the pattern for the other biomaterials. Figure 5 shows the total biofilm mass determined by CV staining. Although the absorbance value for Co-Cr-Mo tended to be lower than for the other materials, there was no significant difference between the materials (*P*>0.05). Correspondingly, the VCC values did not show any significant difference between the five materials (Figure 6) (*P*>0.05).

## Discussion

In this research, we evaluated the difference in early biofilm formation of PIA-positive *S. epidermidis* on five types of biomaterials - including Oxinium, which is now being used as a new material for prosthetic joints. We also investigated the correlation between the physical characteristics of the various biomaterials and their ability to form biofilms at an early stage. After culturing for 2 and 4 hours, biofilms had formed on all the test materials and the BCR values were similar for all of them (*P*>0.05). Previous reports have shown that bacterial adhesion is primarily determined by a threshold surface roughness of Ra more than 0.2 µm (200 nm) [33], [51]. Therefore, we polished the specimen surfaces to similar degree of smoothness (Ra<10 nm) in order to eliminate any discrepancies due to the effect of surface roughness. This high level of surface smoothness is thought to be the reason that no significant difference in BCR was observed between specimens until culturing exceeds 4 hours.

After culturing for 6 hours, Co-Cr-Mo had a significantly lower BCR than Ti-6Al-4V, cp-Ti and stainless steel (*P*<0.05). However, the total biofilm mass determined by CV staining and the viable cell counts did not differ significantly between the materials (*P*>0.05). Boks et al reported that bond strengthening for four strains of *S. epidermidis* on a hydrophobic surface was limited to a minor increase [52]. Tang et al showed that more bacteria adhered to a hydrophilic surface than a hydrophobic surface [53]. As water molecules adjacent to a hydrophobic surface are not able to form hydrogen bonds with that surface (hydrophobic effect), bacterial adhesion to a hydrophobic surface is brought about by an entropically favorable release of water molecules. With regards to surface free energy, numerous studies in the dental field agree that surfaces with high surface free energy foster microbial adherence *in vitro* and *in vivo* [31]–[33], [54]–[56]. Glantz et al reported that when analyzed gravimetrically, there was less dental plaque on low surface free energy hydrophobic substrata than on hydrophilic substrata due to the effect of interfacial thermodynamics [57]. On the other hand, Van Pelt et al suggested that surface free energy is presumably more directly related to the binding force rather than to the number of bacteria on the surface area [54]. Therefore, it can be speculated that bacteria on the relatively hydrophobic Co-Cr-Mo surface, which has the lowest surface free energy, binds cell-to-cell more tightly with polysaccharides than to a cell-to-material surface (bacteriophobic effect), and that it is difficult for bacteria to develop a biofilm on the horizontal plane on the Co-Cr-Mo surface. However, the ability of bacteria to adhere and form a biofilm, as described by Cerca et al, varies to a wide degree depending on the strain of *S. epidermidis* [58]. Schildhauer et al also reported that *S epidermidis* varied in its adherence to various metallic implants and there was no significant difference between them [59]. Thus, the literature does not agree on how the physical characteristics of a biomaterial influence early biofilm formation. It is also possible that additional physico-chemical characteristics, such as released metal ions and chemical structure, may have some influence that inhibits or delays biofilm development. Poortinga et al showed that the change in substratum potential as a function of the number of adhering bacteria is a measure of the amount of electric charge transferred between the substratum and the bacteria during adhesion [60]. Thus, early biofilm formation is a multi-factorial process that is unlikely to be explained by a single surface characteristic. Further study is needed to refine these results of this study.

Several limitations must be noted in interpreting the data. We established an *in vitro* model that imitates early biofilm growth on the surface of a biomaterial and measured the amount of undamaged biofilm with BCR, the total biofilm mass using the CV assay and the number of viable bacteria in the biofilm using VCC. However, we cannot deny the possibility that the polishing and washing processes may have affected the surface physical characteristics of the biomaterials. Although the complex phenomena that occur *in vivo* were not accurately reproduced, a simple comparison of biofilm formation capability on various material surfaces can be made. To our knowledge, studies that evaluate the bacteriological characteristics of biofilms on Oxinium have not yet been carried out. This study allowed greater control of the experimental variables and produced fewer artifacts in the results. The ultimate research goal is to identify how the pathogens causing implant-related infections interact with biomaterial surface characteristics to affect the process of biofilm formation. We consider that our study has provided valuable results in the early stages of assessment of biofilm formation. These simple configurations are particularly encouraging as tests for use and have demonstrated that surface wettability and surface free energy have an effect on horizontal expansion in the development of biofilm.

## Conclusions

We compared early biofilm formation ability on the surface of five types of solid biomaterials, eliminating the effect of surface roughness on the process. After culturing for 2 and 4 hours, there was no significant difference in the BCR of the five materials. After culturing for 6 hours, the BCR for Co-Cr-Mo alloy was significantly lower than that for Ti-6Al-4V, cp-Ti and stainless steel. However, while the absorbance value determined by the crystal violet assay and the number of colony forming units calculated by a viable cell count tended to be low for Co-Cr-Mo alloy, there was no actual significant difference. These results suggest that surface characteristics, mainly wettability and surface free energy, may have some effect on horizontal expansion in biofilm development.
